# Resource flows and levels of spending for the response to HIV and AIDS in Belarus

**DOI:** 10.1186/1756-0500-4-248

**Published:** 2011-07-21

**Authors:** Valentina I Kachan, Alena I Tkachova, Eleanora Gvozdeva, Ilona Urbanovich, Anna Yakusik, Peter Amico, Carlos Avila-Figueroa

**Affiliations:** 1 Deputy Minister of Health, Ministry of Health, Miasnikov Street 39, Minsk, 220048, Belarus; 2 Head of the Health Care Planning and Financing Department, Ministry of Health, Miasnikov Street 39, Minsk, 220048, Belarus; 3 Country Officer, UNAIDS, Krasnoarmieskaya Street 22a, Minsk, 220050, Belarus; 4 Consultant, UNAIDS, Krasnoarmieskaya Street 22a, Minsk, 220050, Belarus; 5 Heller School for Social Policy and Management, Brandeis University, 415 South Street Waltham, MA, 02454, USA; 6 Economics and Financing Division, UNAIDS, Avenue Appia 20 Geneva, 1211, Switzerland

## Abstract

**Background:**

Belarus has a focused HIV epidemic concentrated among injecting drug users, female sex workers and men who have sex with men. However, until 2008, Belarus had no way of evaluating HIV spending priorities. In 2008, Belarus committed to undertaking a comprehensive National AIDS Spending Assessment (NASA) in order to analyze HIV spending priorities. NASA was used to 'follow the money' from the funding sources to agents and providers, and eventually to beneficiary populations.

**Findings:**

Belarus spent the majority of its funding on prevention, diagnosis and treatment of sexually transmitted infections and on securing the blood supply. International donors and NGOs working within Belarus spent the majority of their funding on preventative activities for high risk groups while Global Fund to Fight AIDS, Tuberculosis, and Malaria (GFATM) solely funded antiretroviral treatment.

**Conclusions:**

The data and experience obtained through conducting NASA will help build capacity for future resource tracking activities for HIV and other health priorities. This experience established the foundation for enhanced and future consistent quality-reporting of National Health Accounts. Monitoring the flow of resources for Belarus' HIV response provides valuable strategic information that can improve operations and planning as well as mobilize greater resources. NASA offers Belarusian policy makers an overview of HIV activities that merit their priority attention. In addition, the findings from Belarus are particularly relevant for the rest of the Commonwealth of Independent States due to their similar epidemiological profiles and centrally planned systems. The Belarusian government faces future challenges, especially in increasing public investments in HIV prevention for female sex workers and their clients, men who have sex with men, and among intravenous drug users.

## Background

Belarus was minimally impacted by HIV until 1996-1997, when an HIV outbreak in Svetlogorsk increased the number of infected individuals by more than tenfold [1], since then, a total of 10,690 cases of HIV have been registered with an estimated 17,000 cases as of 2009 [2]. The country still has a relatively low prevalence rate (0.2-0.3%); the epidemic is concentrated among injecting drug users (IDUs), female sex workers (FSW) and men who have sex with men (MSM) with 13.7, 2.7 and 6.4 percent prevalence rates respectively [2,3]. Estimates of the number of IDUs in the country range from 62,000 to 77,000 [4,5]. The majority of HIV positive people are between the ages of 15-29 [5].

Belarus, an upper-middle income country [6] with a population of 9.6 million, is located in the center of Europe and is surrounded by Russia, Ukraine, Poland, Lithuania and Latvia [5]. The country has no access to the sea, but it is located in an important location, facilitating trade and travel between Europe and the Commonwealth of Independent States (CIS) [5]. In 2008, GDP was $US 60.343 billion with a ten percent growth rate from the previous year [5]. 6.5 percent of GDP was spent on public health including 3.6 percent of government spending and 2.9 percent of non-governmental (NGO) spending.

The literature has suggested that countries will have to make hard decisions about where to allocate HIV funding due to impending financing shortages [7,8]. For this reason, Belarus committed to undertake a form of resource tracking in order to better understand the spending patterns for HIV treatment and HIV related activities. With this information, they hoped to be able to make more informed decisions about funding allocation as well as anticipating future threats to sustainability. In 2008, Belarus implemented their first comprehensive National AIDS Spending Assessment (NASA) of public, international and private HIV-related expenditures. Using the most recently available data, this paper describes HIV spending patterns in Belarus and their implications for future policy decisions.

## Methods

A comprehensive tracking and analysis of HIV expenditures in 2008 was conducted by training and overseeing resource tracking teams covering each one of the six voblasts and Minsk; Minsk capital, Brest, Gomel, Grodno, Minsk Region, Mogilev and Vitebsk. A review of key policy documents, program documentation and budgetary expenditure reports was performed using standard methodology, formats and definitions [5]. All resource tracking teams were trained on using the standard NASA framework to ensure accuracy. Adoption of the official AIDS spending report by the Ministry of Health made it possible to include all of the healthcare organizations in the country [5]. All expenditures, by programmatic activity and HIV services, were cross-tabulated by source of financing and stratified by income level using the NASA framework.

NASA is a tool developed by UNAIDS to measure the entirety of resources included in a country's national HIV response; it was developed using the national health accounts framework and principles [9,10]. NASA applies standard accounting methods to reconstruct all transactions in a given country, 'following the money' from the funding sources to agents and providers, and eventually to beneficiary populations. The NASA methodology was approved by the UNAIDS Global Consortium of Resource Tracking in 2006 and has been used to report progress on the 2001 Declaration of Commitment from the UN General Assembly Special Session on HIV/AIDS (UNGASS). It additionally supports countries in planning and monitoring their HIV activities.

NASA analyses include levels and patterns of domestic HIV spending from public and international sources down to the recipient population. HIV spending is structured into eight categories of spending: (1) prevention; (2) treatment and care; (3) orphans and vulnerable children; (4) program management and administration; (5) human resources; (6) social protection; (7) enabling environment; and (8) research. NASA spending categories are also divided into a functional classification that includes health and non-health HIV services [11].

The NASA process and collection of spending flows requires significant collaboration among government agencies and international organizations. In Belarus, these organizations included the following: the Ministry of Health of Belarus, the National Center for Hygiene, Epidemiology and Public Health, projects of the United Nations Development Program, The Global Fund to fight AIDS, Tuberculosis and Malaria (GFATM), UNAIDS and various NGOs. The UNAIDS country coordinator worked closely with the local NASA team to organize the NASA process in country.

## Results

In 2008, Belarus spent more than US$ 19 million on HIV/AIDS with public service providers accounting for 82 percent (US$ 15.6 million) of the total spending (Table 1). International service providers (NGOs and multi-lateral organizations) represented 10 percent (US$ 1.9 million) of the total with private out-of-pocket expenditures on condoms comprising the remaining 8 percent (US$ 1.5 million). The sources of financing were comprised of 68 percent public funds (US$ 12.96 million), 22 percent international funds (US$ 4.28 million) and 10 percent private funds (US$ 1.86 million). Prevention spending was over 70 percent of the total spending for HIV in 2008; and, of the US$ 13.5 million spent on prevention, almost US$ 10.3 million originated from government expenditures. Intervention areas receiving the next highest amount of contributions included care, and treatment as well as program management, which accounted for 12.9 and 8.6 percent of total expenditures respectively. Due to the high amount of expenditures on prevention, it is constructive to look at a breakdown of prevention spending to assess both country and international priorities.

**Table 1:** Spending on HIV/AIDS.

	Private	Percent Private	Public	Percent Public	International (NGO/Multi-Lateral)	Percent International	Total
**Sources of Finance**	4,275,076	10%	12,956,893	68%	12,956,893	22%	19,095,705

							

**Intervention Areas by Service Provider**							

Prevention	1,514,216	11%	10,973,620	81%	1,062,269	8%	13,550,105

Care and Treatment	-		2,298,965	93%	164,178	7%	2,463,143

Orphans and Vulnerable Children (OVC)	-		93,995	100%	-	0%	93,995

Program Management	-		1,035,586	63%	608,810	37%	1,644,396

Human Capital	-		501,418	89%	64,927	11%	566,345

Social Protection and Social services (excluding OVC)	-		326,173	100%	-	0%	326,173

Enabling environment and community development	-		5,898	15%	33,629	85%	39,527

HIV-related research (excluding operations research)	-		411,553	100%	468	0%	412,021

**Total**	**1,514,216**	**8%**	**15,647,208**	**82%**	**1,934,281**	**10%**	**19,095,705**

**Figure 1:**
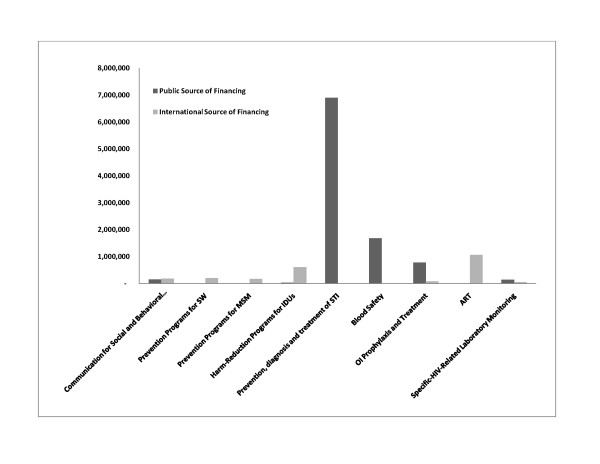
**Key Spending Areas in Prevention and Treatment and Care**.

While prevention is clearly a priority, it is necessary to evaluate if current spending is targeting the most at-risk populations. Figure 1 provides a glimpse of some of the highest spending categories within prevention/care and treatment broken down by government funding and international funding. This figure elucidates the key priorities of the Belarusian government which include prevention, diagnosis and treatment of sexually transmitted infections [12] and investments in blood safety. In contrast, programs for FSWs, MSM, and IDUs are supported primarily by international funding. These represent a low percentage of the overall spending on prevention.

The anti-retroviral drug acquisition [13] program is completely supported by GFATM. Prophylaxis and treatment of opportunistic infections, specific HIV-related laboratory monitoring and communication for social and behavioral change were also financed from both the state budget and international funds (GFATM) [5].

Though out-of-pocket private spending covered payment for condoms, total out-of-pocket expenditures on HIV and AIDS may be underestimated. Household spending for any other forms of prevention or treatment was not captured in this study and thus also may be underestimated.

## Discussion

While HIV spending in Eastern Europe has significantly increased in recent years, this is the first comprehensive report analyzing data on HIV spending from one of the Commonwealth of Independent States (CIS) [14]. The resource tracking system in place documented that Belarus spent more than US$ 19 million on the AIDS response in 2008. While treatment and care represents about 13 percent of the total, more than 70 percent of expenditures were channelled to preventive activities. Once adjusted for the size of the Belarusian population, the per capita spending was US$ 1.98, which is comparable to the average per capita spending on HIV reported from other low and middle income countries [15].

Despite the financial constraints experienced by Belarus, the government has been substantially increasing investments in the social sector, particularly in the area of prevention. Analysis of health expenditures showed per capita spending increasing from US$ 66 in 2000, US$ 147 in 2004 up to US$ 406 in 2008 - a six-fold increase in eight years [16]. Belarus also was the first country in the CIS to reverse overall economic decline with sizeable growth rates averaging 9 percent per annum between 2003 and 2008; during this time period, per capita income tripled, delivering one of the highest poverty rate reductions in the region [16]. The current spending patterns from public sources reflect the emphasis of HIV programmes based on health facilities as 63% of the resources were directed to two interventions; STI treatment and blood safety.

High rates of STI in Eastern Europe are a major public health concern and a potential risk factor for HIV transmission. Case reports and service utilization for STIs are indirect proxies of unsafe sex. Relevant figures for *C. trachomatis *and *N. gonorrhoeae *infections have been reported in the region [17]. The presence of an untreated ulcerative or non-ulcerative infection increases the risk of both acquisition and transmission of HIV. Thus, investments in diagnosis and treatment for STIs are important to reduce the risk of HIV infection. A third of the total spending in prevention was allocated to STIs programmes in Belarus. This funding supported the creation of a medical specialist's task force on STI directing prevention activities among female sex workers and MSM in the major Belarusian cities (Minsk, Brest, Vitebsk, Grodno, Gomel, Mogilev and Svetlogorsk) [18]. Work on prevention of HIV and STIs has been integrated into counselling centers complemented by community outreach workers; condoms, testing and treatment of STIs, and psychological support are provided. Services for sex workers and MSM account for an annual average of 3,000 consultations, 600 STI treatments, almost one million condoms provided and 30,000 printed materials distributed [18].

Screening all donated blood for HIV in accordance with minimum quality standards remains vital. Progress has been made toward eliminating blood transfusion as a significant cause of HIV infection in the region. However, the number and percentage of donations screened in Eastern Europe and Central Asia is relatively low, reported to be 76%, albeit with important regional variation [19]. In Belarus, as the allocation of resources shows and consistent with the country report, 100% of donated blood units are screened for HIV in a quality assured manner [18]. Blood safety programmes are a priority in Belarus and a significant amount of resources support these programs.

Total care and treatment spending was US$ 2.5 million (13% out of the total) and includes provider initiated testing and counselling, treatment and prophylaxis for opportunistic infections, antiretroviral therapy, laboratory monitoring for 1,250 PLWH as well as palliative care [20]. Almost 50% of these resources were spent on ARV medicines and an additional 10% in laboratory monitoring. Most of the resources for treatment and care were funded by international sources (55%) and an interrupted supply of ARVs is possible under a completely GFATM- funded project.

Providers of care are also important partners in the response to HIV. In Belarus, the providers are Ministry of Health facilities, department facilities including hospitals and clinics, multilateral agencies, NGOs and private facilities. Hospitals and clinics play key roles in providing care and prevention, but they could also find innovative ways to create enabling environments and some social services at limited additional cost. The efforts must not be completely top-down, but rather allowing local creativity to innovate and work within a fixed budget.

Monitoring the flow of resources for the HIV response provides valuable information for improving operations and planning, and mobilizing greater resources. NASA offers Belarusian policy makers an overview of HIV activities requiring their attention. It emphasizes issues relevant to the effective use of resources within the health sector. For example, it has been documented that harm reduction activities among intravenous drug users can be cost-effective. A recent study showed cost-effectiveness of US$ 359 per HIV infection averted in Svetlogorsk, Belarus [21]. The same study also showed how relatively small shortfalls in funding can reduce impact and cost-effectiveness. Yet, Belarus is among the few countries of the CIS who realize the need to prioritize resources among groups where a sizable funding gap exists. The mapping of HIV expenditures provides crucial guidance for the reallocation of resources while supporting evidence-based decision making.

An uncontrolled, concentrated epidemic, if left alone has the potential to turn into a generalized epidemic. Increased public investment in HIV prevention for high risk groups including FSWs and their clients, MSM, and especially IDUs, is critical to containing the epidemic. Without any targeted intervention, it is likely that HIV prevalence will continue to increase among these groups in Belarus [5]. Finally, it is necessary to ensure that there is active dialogue and consultation with civil society, including the private sector, on the reform program. Both country health priorities for immediate response as well as longer-term measures must be discussed.

## Conclusion

The data and experience obtained through conducting NASA for 2008 will help build capacity for future resource tracking activities for HIV and other health priorities. This experience established the foundation for enhanced and future consistent quality-reporting of National Health Accounts. This newly laid foundation with improved quality-reporting will enable implementation and regular improvement of the monitoring of the national response to HIV and tracking the efficiency of HIV-related programs and activities. Additionally, it will serve as a basis for improving national strategic planning in health.

The CIS was created a successor entity to the Union of Soviet Socialist Republics (USSR). Many of the current members are from centrally planned economies and therefore have health systems that are similar to Belarus. In Eastern Europe, they key modes of HIV transmission are injecting drug use and sex work [22]. Due to the similar epidemiological profiles of the CIS countries they can benefit from the knowledge gained in Belarus. One of the key lessons for these countries is the importance of increased targeted prevention efforts at high risk groups, especially injecting drug users.

The results from the current AIDS resource tracking exercise in Belarus have been an extremely useful exercise which has generated evidence to guide future policy decisions. The government of Belarus is contributing to the majority of the expenditures for HIV and is the driving force behind prevention. As the present study demonstrates, the majority of prevention spending has been focused on diagnosis and treatment of STIs in the general population while another significant portion of the funding is focused on securing a safe blood supply. The government faces future challenges, especially in increasing public investments in HIV prevention for female sex workers and their clients, men who have sex with men, and intravenous drug users. NASA has helped establish current resource allocations and highlighted the critical role of these investments in containing the HIV epidemic in Belarus.

## Competing interests

The authors declare that they have no competing interests.

## Authors' contributions

VK participated in the design, conception and analysis of the study, AT participated in the design of the study, EG participated in the analysis of the study, IU participated in the analysis of the study, AY participated in the design of the study, PA drafted the manuscript and participated in the analysis and CA drafted the manuscript and participated in the design and analysis of the study. All authors read and approved the final manuscript.
